# High-dose chemotherapy with autologous haematopoietic stem cell transplantation in patients with isolated vitreoretinal lymphoma: a LOC network study

**DOI:** 10.1038/s41409-024-02477-y

**Published:** 2024-11-19

**Authors:** Adam Mainguy, Carole Soussain, Valérie Touitou, Amin Bennedjai, Laurent Kodjikian, Hervé Ghesquières, Gandhi Damaj, Rémy Gressin, Jean-Baptiste Ducloyer, Olivier Chinot, Anaïs Vautier, Cécile Moluçon-Chabrot, Guido Ahle, Luc Taillandier, Jean Pierre Marolleau, Adrien Chauchet, Fabrice Jardin, Nathalie Cassoux, Denis Malaise, Adélaïde Toutée, Sara Touhami, Magali Le Garff-Tavernier, Khê Hoang-Xuan, Sylvain Choquet, Caroline Houillier

**Affiliations:** 1 Sorbonne Université, Department of Ophthalmology, Pitié-Salpêtrière University Hospital, Paris, France; 2 Angers University, Department of Ophthalmology, Angers University Hospital, Angers, France; 3 Department of Hematology, Institut Curie, site de Saint-Cloud, Saint-Cloud, France; 4 Department of Ophthalmology, Lyon University Hospital, Lyon, France; 5 Department of Hematology, Lyon University Hospital, Lyon, France; 6 Department of Hematology, Caen University Hospital, Caen, France; 7 Department of Hematology, Grenoble University Hospital, Grenoble, France; 8 Nantes University, CHU Nantes, ophthalmology department, Inserm, CIC 1413 Nantes, France; 9 Department of Neurooncology, Marseille, University Hospital, Marseille, France; 10 Department of Hematology, Clermont-Ferrand University Hospital, Clermont-Ferrand, France; 11 Department of Neurology, Hospices Civils de Colmar, Colmar, France; 12 Department of Neurology, Nancy University Hospital, Nancy, France; 13 Department of Hematology, Amiens University Hospital, Amiens, France; 14 Department of Hematology, Besançon University Hospital, Besançon, France; 15 Department of Hematology, Centre Henri Becquerel, Rouen, France; 16 Department of Ophthalmology, Institut Curie, site de Saint-Cloud, Saint-Cloud, France; 17 Department of Hematology, Pitié-Salpêtrière University Hospital, APHP, Sorbonne Université, Paris, France; 18 Department of Neurooncology, Pitié-Salpêtrière University Hospital, APHP, Sorbonne Université, Inserm, CNRS, UMR S1127, ICM, IHU, Paris, France; 19 Department of Clinical Hematology, Pitié-Salpêtrière University Hospital, APHP, Sorbonne Université, Paris, France

**Keywords:** Stem-cell therapies, Disease-free survival

## Abstract

Despite its indolent evolution, vitreoretinal lymphoma (VRL) has a poor prognosis due to a major risk of relapse in the central nervous system (CNS) and may necessitate aggressive therapy. However, the use of high-dose chemotherapy with autologous stem cell transplantation (HCT-ASCT) is poorly documented. We retrospectively analysed from the French LOC network database the adult immunocompetent patients treated with HCT-ASCT for isolated VRL. Thirty-eight patients underwent consolidation with HCT-ASCT for isolated VRL between 2008 and 2019 after induction chemotherapy. Twenty patients had primary VRL, and 18 had an isolated VRL relapse of a primary CNS lymphoma. Three patients underwent HCT-ASCT in first-line treatment, 24 in second-line treatment, and 11 in subsequent lines. At HCT-ASCT, the median age was 61 years, and the median KPS was 90. Thirty-two patients (84%) received high-dose thiotepa-based HCT. One patient (3%) died from HCT-ASCT toxicity. Nineteen (50%) patients relapsed after HCT-ASCT, including 17 cases occurring in the brain. The median progression-free survival, brain-free survival and overall survival from HCT-ASCT were 96, 113 and 92 months, respectively. HCT-ASCT represents an effective therapeutic strategy for select VRL patients, with a tolerable safety profile. However, the risk of subsequent brain relapse remains significant.

## Introduction

Vitreoretinal lymphoma (VRL) is a rare type of lymphoma, most often of the diffuse large B-cell type [1]. Patients have a median age of 70 years at the time of diagnosis [2]. VRL can be either isolated (primary VRL [PVRL]), isolated intraocular relapse of primary central nervous system lymphoma (PCNSL) or exceptionally isolated intraocular relapse of systemic lymphoma or associated with PCNSL, the latter in 10–20% of cases [3–6].

Isolated VRL is an indolent disease that often causes few or no symptoms, and it typically progresses slowly without treatment, in contrast to the highly symptomatic and rapidly progressing nature of PCNSL. This indolent progression contrasts with a poor long-term prognosis due to the significant risk of developing central nervous system lymphoma (CNSL), which has a worse prognosis. Forty to 90% of patients with PVRL develop central nervous system (CNS) disease after 30 months of follow-up [1, 7–9]. Patients with VRL associated with CNS involvement have a poor prognosis, with a median overall survival (OS) of 18–34 months [8, 10, 11], compared to 37–75 months for patients with isolated VRL [2, 7, 12].

Like the brain, the eye is considered an immune-privileged site isolated from the remaining blood system [13], which represents a therapeutic challenge. The treatment of VRL remains controversial and poorly standardized due to its rarity and the absence of prospective studies [1, 14, 15]. Consequently, a multidisciplinary approach involving ophthalmologists, haematologists, and neurologists is needed. The optimal treatment for relapsing VRL, in the absence of cerebral involvement, is even more controversial. Therapeutic strategies range from local intraocular treatments to more aggressive treatments with several systemic chemotherapies [1, 10, 12, 14]. The choice of systemic treatment is based on an analogy with what is done in PCNSL, with the frequent use of high-dose methotrexate [1, 2].

Despite the rather grim disease prognosis, there are limited data available in the literature regarding HCT-ASCT in the context of VRL [16–19], and to our knowledge, there is no dedicated study on HCT-ASCT in the context of isolated VRL. In France, HCT-ASCT appears to be a justified treatment for VRL, given the unfavourable prognosis of the disease, and it has been included in the treatment recommendations for PVRL since the end of the 2000s. Therefore, the aim of this study was to retrospectively review a national case series of VRL patients treated with consolidative HCT-ASCT to assess the efficacy and safety of this therapeutic strategy.

## Patients and methods

This work is based on the analysis of the French LOC (“Lymphomes Oculo-Cérébraux”) network database, a nationwide database centralizing information from 28 different centres in France, representing the main centres involved in PCNSL management. The database was approved by the institutional ethical committee of the coordinating centre and by the French data protection agency (CNIL: Commission Nationale de l’Informatique et des Libertés). All patients provided informed consent to participate in the database and for the use of their data. The study was carried out following the tenets of the Declaration of Helsinki.

Patients were retrospectively selected from the LOC database according to the following criteria: 1) isolated vitreoretinal lymphoma at the beginning of the line of treatment when HCT-ASCT was performed: either primary VRL or isolated intraocular relapse of either primary VRL or PCNSL. Cerebral and cerebrospinal fluid (CSF) involvement had to be excluded by cerebral MRI and lumbar puncture. Patients with isolated vitreoretinal relapses of systemic lymphomas were excluded from the present work; 2) HCT-ASCT applied as a consolidation treatment after induction therapy at any line of treatment; 3) aged >18 years; 4) immunocompetent status; 5) pathological diagnosis or cytological diagnosis in the vitreous at initial diagnosis. Patients were selected in January 2020, and data were analysed in October 2023.

The main outcome endpoints were response after HCT-ASCT, OS, progression-free survival (PFS), brain-free survival (BFS) and toxicity of HCT-ASCT. The response to HCT-ASCT was assessed according to the IPCG criteria with the following criteria: complete response (CR): no evidence of residual disease in the anterior chamber, vitreous or retina; partial response (PR): >50% reduction in ophthalmological findings; progressive disease (PD): worsening of ocular findings or new ocular lesions; and stable disease (SD): none of the previous items [20]. Clinical evaluation was completed by measuring interleukin 6 (IL-6) and 10 (IL-10) levels in the aqueous humour when available. The IL-10 concentration in the aqueous humour was considered elevated, with a cut-off of 30 pg/ml [21]. Depending on the analysis, the endpoints were calculated either from the day of infusion of the stem cells or from the initial diagnosis. PFS was defined as the time without relapse (whatever its location) or without death (whatever its cause). BFS was defined as the time without brain relapse. Survival rates were calculated using the Kaplan‒Meier method. Toxicity was assessed according to the Common Terminology Criteria for Adverse Events version 4. The log-rank test was used to test for the equality of the survival distributions. Two-sided *p* values < 0.05 were considered significant. The statistical analyses were carried out with R software (R Core Team (2022). R: A language and environment for statistical computing. R Foundation for Statistical Computing, Vienna, Austria. URL https://www.R-project.org/.

## Results

### Patient characteristics

Thirty-eight patients (22 females and 16 males) from 15 centres who were diagnosed with CNS DLBCL between 1995 and 2018 and treated with HCT-ASCT between 2008 and 2019 were included in the study. The main characteristics of the patients before HCT-ASCT are indicated in Tables 1 and 2.

**Table 1:** Patient characteristics before HCT-ASCT.

Patient’s characteristics before the line of treatment of HCT-ASCT	
*N*	38
Age at initial diagnosis, median [Min, Max]	59.5 [40–71]
Female gender (*N*, %)	22 (58%)
Localization of initial lymphomatous involvement (*N*, %)	
CNS	11 (29%)
CNS + VR	7 (18%)
VR	20 (53%)
CNS involvement at any time before HCT-ASCT	18 (47%)
Previous treatments (*N*, %)	
HD-MTX	37 (97%)
Rituximab	21 (55%)
HD-AraC	31 (82%)
WBRT	8 (21%)
Refractory after the 1^st^ line of treatment (*N*, %)	11 (29%)

Patient’s characteristics at the beginning of the line of treatment of HCT-ASCT	
Treatment line of HCT-ASCT (*N*, %)	
1^st^	3 (8%)
2^nd^	24 (63%)
>2^nd^	11 (29%)
Characteristics of ophthalmologic involvement (%) at the beginning of the induction treatment before HCT-ASCT	
Unilateral involvement	16/29 (55%)
Visual acuity decrease	20/27 (74%)
Floaters	6/27 (22%)
IL-10 dosage in the anterior chamber at the beginning of the induction treatment before HCT-ASCT	
Number of IL-10 dosages available	22 (58%)
IL-10 level (pg/ml): median (Min-Max)	360 (2–5000)
Elevated IL-10 (>30 pg/ml)	17/22 (77%)
% with a positive ISOLD score (Costopoulos et al. [21])	16/22 (73%)
IL-10/IL-6 ratio (>1)	18/22 (82%)
Induction chemotherapy used before HCT-ASCT (*N*, %)	
Systemic chemotherapy	35 (92%)
(R)-ICE	19/35 (54%)
HD-MTX-based CT	8/35 (23%)
HD-AraC-based CT	6/35 (17%)
Temozolomide	2/35 (6%)
Local treatment (intravitreal MTX)	6 (16%)
Alone	3/6 (50%)
Combined with systemic chemotherapy	3/6 (50%)

*N* number of patients, *%* proportion of patients, *CNS* central nervous system, *VR* vitreoretinal, *HCT-ASCT* high-dose chemotherapy with autologous haematopoietic stem cell transplantation, *HD-MTX* high-dose methotrexate, *HD-AraC* high-dose cytarabine, *WBRT* whole-brain radiotherapy, *IL-10* interleukin 10, *IL-6* interleukin 6, *(R)-ICE* (rituximab)-ifosfamide, carboplatin, etoposide, *CT* chemotherapy.

**Table 2:** Patient characteristics at the time of HCT-ASCT.

*N*	38
Delay between initial diagnosis and HCT-ASCT: median (range), months	21.6 (4.9–116.3)
Delay between isolated ophthalmological relapse (or diagnosis for HCT-ASCT in the 1^st^ line) and HCT-ASCT: median (range), months	4.9 (2.6–7.2)
Age at HCT-ASCT, median [Min-Max]	61 [42–73]
KPS, median [Min, Max] at HCT-ASCT	90 [60,100]
Mean best corrected visual acuity at HCT-ASCT (decimal scale)	6.33 ± 2.26
Tumoral status at HCT-ASCT (N, %)	
Complete response	29 (76%)
Partial response	7 (18%)
Stable disease	1 (3%)
Progressive disease	1 (3%)
IL-10 dosage in the anterior chamber just before HCT-ASCT	
Number of dosages available	13 (34%)
IL-10 level (pg/ml): median (Min-Max)	10 (0–265)
Elevated IL-10 (>30 pg/ml)	5/13 (38%)
IL-10/IL-6 ratio >1	6/13 (46%)

*N* number of patients, *%* proportion of patients, *HCT-ASCT* high-dose chemotherapy with autologous haematopoietic stem cell transplantation, *KPS* Karnofsky Performance Status, *IL-10* interleukin 10, *IL-6* interleukin 6.

At initial diagnosis, 20/38 (53%) patients had PVRL, and 18/38 (47%) had PCNSL (including 7/38 (18%) who also had ocular involvement). At the time of HCT-ASCT, the median age was 61 years (range 42–73 years) and the median KPS was 90 (range 60–100). Three of the 38 (8%) patients received HCT-ASCT in the first-line of treatment; 24/38 (63%), in the second line of treatment; and 11/38 (29%), in subsequent lines of treatment. The induction treatments used before HCT-ASCT consolidation are listed in Table 1. At the time of HCT-ASCT, 29/38 patients (76%) had a complete response (CR), 7/38 (18%) had a partial response (PR), 1/38 (3%) had stable disease (SD) and 1/38 (3%) had progressive disease (PD); additionally, 5/13 patients still had elevated IL-10 levels in the aqueous humour (including 3 patients in CR, 1 in PR and 1 in SD).

**Table 3:** HCT-ASCT and follow-up after HCT-ASCT.

*N*	38
HCT regimens (*N*, %)	
TBC^a^	23 (61%)
TTP-BCNU^a^	7 (18%)
TTP-Bu^a^	2 (5%)
BEAM	6 (16%)
Tumour response after HCT-ASCT (*N*, %)	
Complete response	33 (87%)
Partial response	2 (5%)
Stable disease	0 (0%)
Progressive disease	2 (5%)
Not assessable (toxic death)	1 (3%)
Mean best corrected visual acuity after HCT-ASCT (decimal scale)	8.20 ± 1.72 *p* = 0.001^b^
Location of the first relapse (*N*, %)	
CNS	6/19 (32%)
CNS and ocular	4/19 (21%)
Ocular	8/19 (42%)
Systemic	1/19 (5%)
Location of relapses throughout the disease after HCT-ASCT (*N*, %)	
Only ocular relapse(s)	1/19 (5%)
At least one brain relapse	17/19 (90%)
Systemic, ocular and meningeal relapse	1/19 (5%)
Relapse rate after HCT-ASCT according to the HCT-ASCT treatment line (*N*, %)	
1^st^ line	1/3 (33%)
2^nd^ line	12/24 (50%)
>2^nd^ line	6/11 (55%)
Relapse rate after HCT-ASCT according to the type of HCT (*N*, %)	
TBC or TTP-Bu	11/25 (44%)
TTP-BCNU	4/7 (57%)
BEAM	4/6 (67%)
Brain relapse rate after HCT-ASCT according to the initial localization of the disease (*N*, %)	
PVRL	9/20 (45%)
PCNSL	8/18 (44%)

*N* number of patients, *%* proportion of patients, *TBC* Thiotepa-busulfan-cyclophosphamide; TTP-BCNU: Thiotepa-carmustine; BEAM: Carmustine, etoposide, cytarabine, melphalan; TTP-Bu: Thiotepa, busulfan. HCT-ASCT high-dose chemotherapy with autologous haematopoietic stem cell transplantation, PVRL primary vitreoretinal lymphoma, PCNSL primary central nervous system lymphoma.

^a^Doses of thiotepa in the various thiotepa-based regimen. TBC: thiotepa 250 mg/m2/day (D) at D-9, D-8, D-7. TTP-Bu: up to 65 years: thiotepa 250 mg/m2/D on D-7, D-6, D-5; if >65 years: 5 mg/kg/D on D-5 and D-4. TTP-BCNU: thiotepa 5 or 10 mg/kg/D on D-5 and D-4 depending on whether > or <65 years of age.

^b^Paired Student’s *t* test comparing the mean best corrected visual acuity before and after HCT-ASCT.

### HCT-ASCT and follow-up after HCT-ASCT (Table 3)

Thirty-two of 38 patients (84%) received thiotepa-based HCT. Thirty-three of 38 patients (87%) achieved CR after HCT-ASCT. Among the 33 patients who achieved CR, 10 patients (30%) had aqueous humour IL-10 levels available after HCT-ASCT, with a median IL-10 level of 2.5 pg/ml (range: 0–7). Two patients (5%) experienced progressive disease with active vitritis just after HCT-ASCT (one who was in CR with a normal IL-10 level in the aqueous humour before HCT-ASCT and one with SD and increased IL-10 levels before HCT-ASCT).

**Fig. 1:**
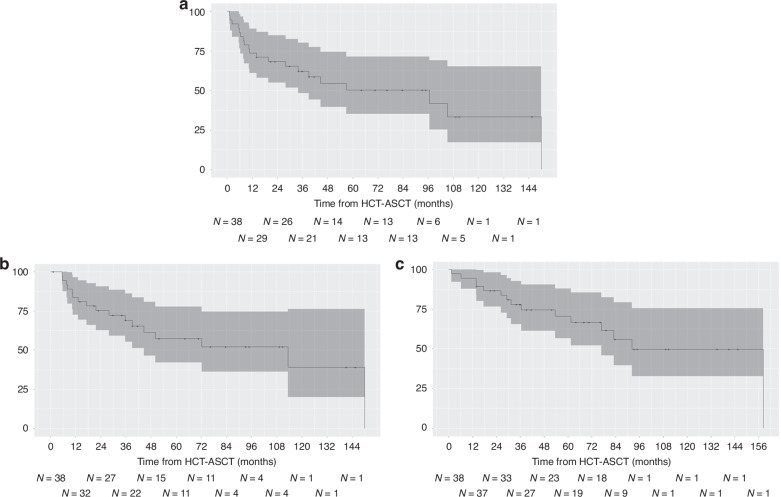
Outcome of patients with VRL treated with HCT-ASCT (**a**: progression-free survival, **b**: brain-free survival, **c**: overall survival). N Number at risk for each timepoint.

The median follow-up from HCT-ASCT was 79 months (95% CI: 64–111). Nineteen of the 38 patients (50%) experienced relapse during the follow-up after HCT-ASCT. There was brain involvement in 10/19 (53%) patients at the 1^st^ relapse following HCT-ASCT. A total of 17/38 (45%) patients (including 9/20 patients (45%) with initial PVRL and 8/18 patients (44%) with initial PCNSL) experienced brain relapse during follow-up. Of the 3 patients who received HCT-ASCT in first-line treatment, one experienced relapse (ocular relapse 56.8 months after HCT-ASCT with BEAM, followed by cerebral relapse 72 months after HCT-ASCT and death at 83 months), while the other 2 patients who received TBC were still disease free at 60 and 109 months. Among the five patients with persistent increased IL-10 levels in the aqueous humour at the time of HCT-ASCT, three relapsed, one died from HCT-ASCT toxicity, and only one did not relapse. The median PFS was 96 months (95% CI: 34-NA). The 2-year and 5-year PFS rates were 68% (95% CI: 59–88) and 50.2% (95% CI: 36–74), respectively (Fig. 1a). In the 35 patients who underwent HCT-ASCT in the setting of relapse, the PFS from HCT-ASCT (2-year PFS: 66% (95% CI: 52–83%)) was significantly longer than the PFS between initial diagnosis and first relapse (2-year PFS: 29% (17–48%)) (*p* < 0.001). The median BFS was 113 months (95% CI: 45-NA). The 2-year and 5-year BFS rates were 75% (95% CI: 59–88) and 57% (95% CI: 36–74), respectively (Fig. 1b).

Fifteen patients (40%) died during follow-up. One patient (3%) died from HCT-ASCT toxicity, and the other 14 (37%) died from cerebral progression of lymphoma. The median OS from HCT-ASCT was 92 months (95% CI: 77-NA). The 2-year and 5-year OS rates were 87% (95% CI: 72–96) and 71% (95% CI: 46–82), respectively (Fig. 1c). The median OS from initial diagnosis was 185 months (95% CI: 83-NA), with a 5-year OS rate of 79% (95% CI: 67–94).

### Toxicity

**Fig. 2:**
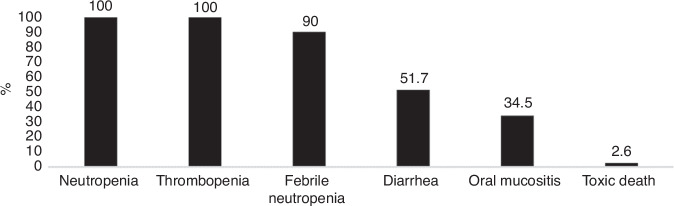
**Grade 3-4 adverse events related to HCT-ASCT**. % proportion of patients.

The grade 3-4 adverse events related to HCT-ASCT are represented in Fig. 2. One hundred percent and 88% of patients experienced grade 4 neutropenia and thrombocytopenia, respectively. The median duration of grade 4 neutropenia was 9 days (range: 3–18). The median duration of grade 4 thrombocytopenia was 4 days (range: 0–83). Data on skin toxicity were lacking. The median hospitalization duration for patients who underwent HCT-ASCT was 21 days (range: 13–129). The data revealed that 19%, 7% and 4% of patients had an in-hospital length of stay exceeding 30, 60 and 90 days, respectively. One patient was hospitalised for 129 days due to the development of multiple complications, predominantly infectious, in conjunction with prolonged cytopenia. One patient died from HCT-ASCT toxicity following acute respiratory distress syndrome after pneumonitis in the context of grade 4 neutropenia, 42 days after HCT-ASCT. He was a 62-year-old patient with a KPS of 80 who received HCT-ASCT (TBC) as a consolidation of 2^nd^-line treatment for ocular relapse of a PCNSL.

### Prognostic factors

No factors, most notably age, KPS, initial diagnosis (PVRL vs. PCSNL) (Supplementary Fig. 1) and line of treatment with HCT-ASCT (Supplementary Fig. 2), were significantly associated with OS according to univariate analysis (Table 4).

**Table 4:** Prognostic factors for overall survival (univariate analysis).

		Univariate analysis			
	*N*	Median OS (months)	Hazard Ratio	95% CI	*p* value
Age at HCT-ASCT ≤ 60 years	18	92.2	1.15	(0.39–3.34)	0.79
Age at HCT-ASCT > 60 years	20	158			
Female sex	22	76.8	0.37	(0.12–1.10)	0.06
Male sex	16	158			
KPS at HCT-ASCT ≤ 80	12	92.2	0.62	(0.21–1.81)	0.38
KPS at HCT-ASCT > 80	26	158			
No initial cerebral involvement	20	92.2	1.29	(0.45–3.70)	0.63
Initial cerebral involvement	18	NA			
HCT-ASCT in the first line	3	83.1			
HCT-ASCT in the 2^nd^ line	24	92.2	2.10	(0.26–16.6)	0.48
HCT-ASCT in the > 2^nd^ line	11	158	1.12	(0.12–10.8)	0.92
Unilateral ocular involvement before HCT-ASCT	16	158	3.26	(0.62–17.1)	0.16
Bilateral ocular involvement before HCT-ASCT	13	92.2			
TBC or TTP-BU conditioning	25	158			
TTP-BCNU conditioning	7	53.6	2.87	(0.82–10.0)	0.10
BEAM conditioning	6	61.6	2.09	(0.53–8.15)	0.29
In CR after salvage chemotherapy	29	92.2	0.96	(0.26–3.48)	0.96
Not in CR after salvage chemotherapy	9	NA			
Elevated IL-10 level before HCT-ASCT	5	53.6	4.45	(0.46–42.8)	0.19
Normal IL-10 level before HCT-ASCT	8	NA			

*N* number of patients, *KPS* Karnofsky Performance Status, *TBC* Thiotepa-busulfan-cyclophosphamide, *TTP-BCNU* Thiotepa-carmustine, *BEAM* Carmustine, etoposide, cytarabine, melphalan, *TTP-Bu* Thiotepa, busulfan, *HCT-ASCT* high-dose chemotherapy with autologous haematopoietic stem cell transplantation, *CR* complete remission.

There was no significant difference in terms of BFS between patients with PVRL (median: 113 months) and patients with PCNSL at initial diagnosis (median: not reached) (*p* = 0.3).

## Discussion

To our knowledge, this work represents the first large study with a long follow-up dedicated to HCT-ASCT in patients with isolated VRL. Autologous stem cell transplantation with a thiotepa-based conditioning regimen has been the standard first-line consolidation treatment for PCNSL, according to two major randomized phase II studies [22–24]. However, this treatment is still underutilized in PVRL, with limited data available in the literature. However, several studies from our team since the 2000s have shown the feasibility and effectiveness of HCT-ASCT for the treatment of VRL. Soussain et al. conducted a pilot study in 1996 involving five patients with refractory PVRL who underwent HCT, demonstrating its feasibility and showing encouraging results, with no cerebral relapse observed during the follow-up period (between 16 and 26 months) [25]. Soussain et al. conducted another study in 2001, which further demonstrated the feasibility of this treatment and revealed promising outcomes (3-year overall survival of 63.7% in 22 patients, including 3 with relapsed VRL and 6 with isolated vitreoretinal relapse of PCNSL) [16]. Subsequently, two additional studies were conducted: there was a 2-year OS rate of 69% after HCT-ASCT in a study of 43 relapsed patients including 5 with VRL [17], and a median OS of 86 months after HCT-ASCT in 19 patients with relapsed VRL [26]. More recently, Lee et al. described two patients with VRL treated with first-line HCT-ASCT who achieved long-term survival with follow-up durations of 7 and 8 years, respectively [27].

The results observed in our study were generally favourable (median OS, BFS, and PFS from HCT-ASCT of 92, 113 and 96 months, respectively). The PFS from HCT-ASCT was much longer than the PFS from initial diagnosis. These results represent a better prognosis than that usually described in the literature for PVRL (median overall survival and progression-free survival from initial diagnosis ranging from 37 to 75 months [2, 10, 12] and from 18 to 46 months [2, 11, 12, 28, 29], respectively, in the main studies, with heterogeneous treatments ranging from local treatments to systemic treatment mainly based on high-dose methotrexate or to combined systemic and local treatments) as well as for isolated ocular relapses of PCNSL (median overall survival and median progression-free survival from the date of isolated ocular relapse of 57.1 months and 12.2 months, respectively) [19]. We can reasonably imagine that most of the patients who were still disease-free 5 years after HCT-ASCT were definitely cured with this strategy. However, these results must be interpreted cautiously, especially given the retrospective nature of this study, with a high proportion of young patients having a good KPS, which is a factor well known to be associated with a better prognosis in PCNSL [5, 30, 31].

Most of the patients in our study received a thiotepa-based HCT. Due to the limited number of patients, it was difficult to compare the effectiveness of the various regimens or of thiotepa-based HCT versus BEAM. However, with 4 of 6 patients who relapsed following BEAM and given the poor results of this regimen in PCNSL cases [18, 32], our recommendation would be to prefer thiotepa-based regimens.

Despite the favourable survival outcomes, the post-HCT-ASCT relapse rate, particularly the cerebral relapse rate, remains high, both in patients with PVRL and in patients with PCNSL at initial diagnosis (45% at 5 years, 45% in patients with initial PVRL, 44% in patients with initial PCNSL). An important issue that could partly explain this high rate of relapse is the difficulty in adequately evaluating the quality of response in VRL patients. Currently, according to the IPCG criteria [20], the response to treatment is assessed by a rather subjective clinical ophthalmological evaluation. There is no measurable mass in VRL, and many patients maintain residual abnormalities that may be either only a scar or the persistence of an active disease [33]. We could therefore hypothesize that some patients from our cohort did not exhibit a good response at the time of HCT-ASCT, which is a well-known adverse prognostic factor regarding the efficacy of HCT-ASCT in PCNSL [34]. The systematic assessment of IL-10 in the aqueous humour might be an interesting tool [1]. This marker may have a greater sensitivity than clinical examination in detecting relapses early, although further research is needed to confirm this issue. In our cohort, we had limited data, with only 13 patients whose IL-10 levels were measured prior to HCT-ACST. However, three of five patients with elevated IL-10 levels at the time of HCT-ASCT subsequently relapsed.

The majority of the patients in our series received HCT-ASCT in second line or subsequent lines of treatment. In PCNSL patients, the relapse rate is low following HCT-ASCT performed as a 1st-line treatment (5-year PFS of approximately 75%) [18] but is significantly higher in the setting of relapse, with rates close to those observed in our series (2^nd^-line treatment: 5-year PFS of 48% in PCNSL patients [18] vs. 44% in VRL patients in our series). In our cohort, only three patients received first-line HCT-ASCT, two of whom never relapsed after 5 and 9 years of follow-up. However, this represents a cohort that was too small to draw any adequate conclusions. Considering the similarity of the results observed in PCNSL and in PVRL, we could hypothesize that the results of HCT-ASCT in the 1st-line treatment of PVRL would be better than those in the setting of relapse. In light of the poor long-term prognosis of this disease, the use of HCT-ASCT as a 1st-line treatment for VRL should be discussed, especially considering the fact that most PVRL patients experience relapse after “conventional” first-line treatments (with a 5-year PFS of approximately 25% [2, 12]). Given the good KPS of most PVRL patients, this strategy could be used in many of these individuals up to a fairly advanced age, as is done in select elderly patients with PCNSL [22, 35, 36].

This strategy using HCT-ASCT had significant toxicity, but the toxicity profile observed was close to what is usually observed in other studies involving HCT-ASCT [18, 23, 37]. There was a 3% rate of toxicity-related mortality, which is slightly lower than the 5 to 10% range reported for PCNSL [17, 23, 26, 38]. Even if this toxic mortality rate appears to be acceptable, HCT-ASCT remains a toxic treatment that is difficult for many patients to tolerate, and patients should be aware of the limited but real risk of toxic death. Optimizing induction treatments for PVRL, potentially through the incorporation of novel drugs (imids, iBTK, etc.) [39–41], could limit the need for HCT-ASCT by increasing the rate of complete response. We also currently lack molecular prognostic factors that could indicate whether patients belong to a group with a higher or a lower risk of relapse. Several recent studies have revealed promising prognostic molecular factors in PCNSL cases [42, 43], but reliable tools for identifying these factors in patients with VRLs are lacking. There might also be a role for the use of CAR T-cells, which has yet to be determined. Indeed, in systemic lymphomas, therapeutic strategies using CAR T-cells compare favourably to standard strategies, including HCT-ASCT [44, 45]. Promising preliminary data on CNS lymphoma treated with CAR T-cells have been published in recent years, but there are limited data regarding ophthalmological involvement [46, 47].

## Conclusion

Intensive chemotherapy followed by autologous stem cell transplantation is an aggressive therapeutic approach for VRL but appears to provide interesting efficacy results for select patients, with a tolerable safety profile. However, the relapse rate, notably in the brain, following such a strategy remained high in a cohort of patients who mainly received 2^nd^ or subsequent lines of treatment with HCT-ASCT. Several options might improve the prognosis: using HCT-ASCT in 1^st^-line treatment, as in PCNSL, improving the quality of the response prior to HCT-ASCT by incorporating new drugs into the induction chemotherapy regimen or using new strategies such as treatment with CART-cells.

## Supplementary information

Supplemental figure

## Data Availability

All data generated or analysed during this study are included in this published article and its supplementary information files.
