# Supplementary material for: High-dose chemotherapy with autologous haematopoietic stem cell transplantation in patients with isolated vitreoretinal lymphoma: a LOC network study
Source: Bone Marrow Transplant. 2024 Nov 19;60(3):297–304. doi: 10.1038/s41409-024-02477-y (PMC11893470; doi:10.1038/s41409-024-02477-y)

Supplemental figure 1. Overall survival from HCT-ASCT according to initial localization of the disease

(no statistically significant difference between groups p=0.6)


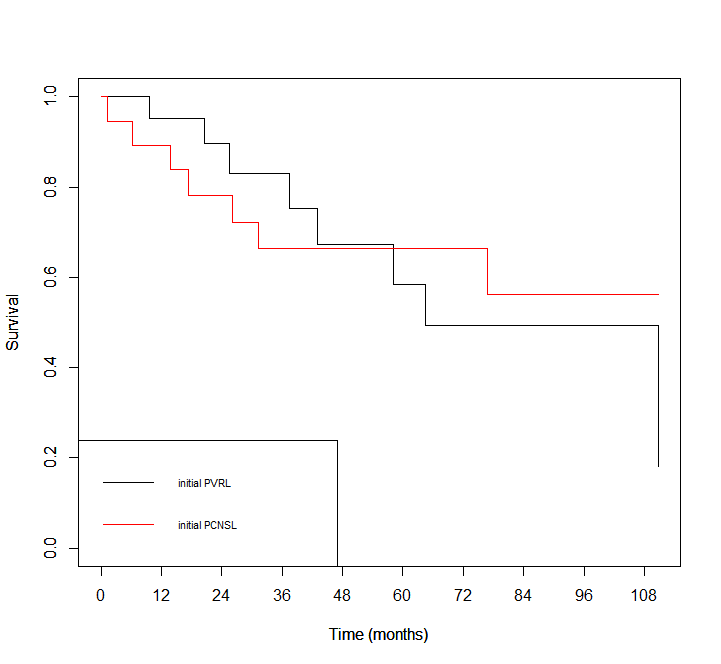


Supplemental figure 2. Overall survival from HCT-ASCT according to the line of treatment

(no statistically significant difference between groups p=0.5)


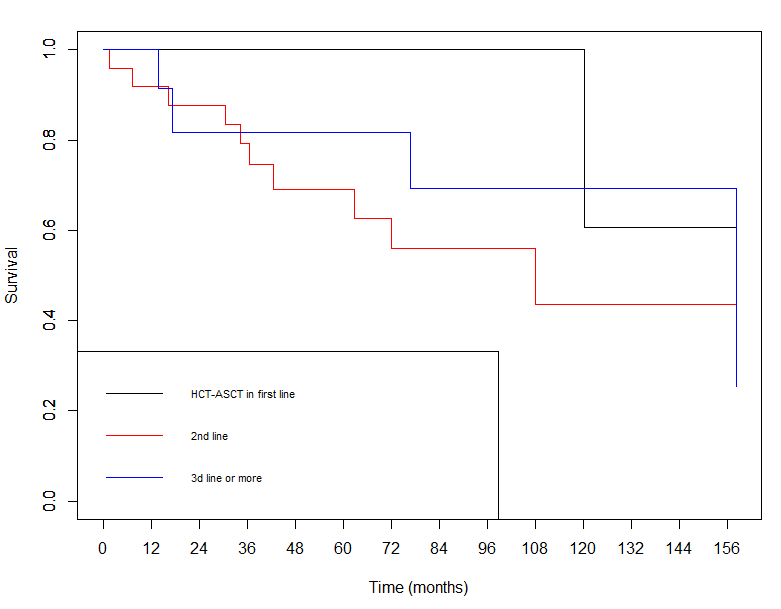

Supplement: Supplementary file 1 — Supplemental figure [file 41409_2024_2477_MOESM1_ESM.docx]
